# Interferon Production and Signaling Pathways Are Antagonized during Henipavirus Infection of Fruit Bat Cell Lines

**DOI:** 10.1371/journal.pone.0022488

**Published:** 2011-07-19

**Authors:** Elena R. Virtue, Glenn A. Marsh, Michelle L. Baker, Lin-Fa Wang

**Affiliations:** 1 Australian Animal Health Laboratory, CSIRO Livestock Industries, Geelong, Australia; 2 Australian Biosecurity Cooperative Research Centre for Emerging Infectious Diseases, Brisbane, Australia; 3 Department of Microbiology and Immunology, The University of Melbourne, Melbourne, Australia; University of Georgia, United States of America

## Abstract

Bats are natural reservoirs for a spectrum of infectious zoonotic diseases including the recently emerged henipaviruses (Hendra and Nipah viruses). Henipaviruses have been observed both naturally and experimentally to cause serious and often fatal disease in many different mammal species, including humans. Interestingly, infection of the flying fox with henipaviruses occurs in the absence of clinical disease. The extreme variation in the disease pattern between humans and bats has led to an investigation into the effects of henipavirus infection on the innate immune response in bat cell lines. We report that henipavirus infection does not result in the induction of interferon expression, and the viruses also inhibit interferon signaling. We also confirm that the interferon production and signaling block in bat cells is not due to differing viral protein expression levels between human and bat hosts. This information, in addition to the known lack of clinical signs in bats following henipavirus infection, suggests that bats control henipavirus infection by an as yet unidentified mechanism, not via the interferon response. This is the first report of henipavirus infection in bat cells specifically investigating aspects of the innate immune system.

## Introduction

Bats have been identified as the natural reservoir hosts of many human and animal pathogens of medical importance, including a spectrum of infectious zoonotic agents such as Ebola virus [1], SARS coronavirus [2], [3], Nipah virus [4], [5] and Hendra virus [6]. Bats are considered one of the more ancient mammals, but little is known about their immune system and how they manage infections. Although bats harbour a large number of emerging pathogens, they rarely show any signs of disease [1], [7], [8], [9], [10], [11]. An example of this is Hendra virus in Australian fruit bats; there is high seroprevalence in the absence of clinical signs suggesting that there is no disease in bats associated with this virus. The nature of persistent, non-clinical, infections and the mechanism of transmission of viruses between bats and from bat to humans and other mammals remains largely unknown. The ability of bats to harbour highly pathogenic viruses, in the absence of significant clinical disease or pathology is driving research into further understanding bat biology, immunology and ecology.

Hendra virus (HeV) and Nipah virus (NiV), from the genus *Henipavirus*, are highly pathogenic viruses that are harboured in pteropodid bats. HeV has occasionally been observed to transmit to and cause disease in horses and then have the ability to infect humans [12], [13], [14], [15], [16], [17], [18], [19]. The single NiV outbreak in Malaysia (NiV-M) occurred following spill-over of virus from bats to pigs, with subsequent transmission from pigs to pig handlers, but no evidence of human to human transmission [20], [21], [22], [23], [24]. The situation in Bangladesh (NiV-B) is different with direct bat to human transmission of NiV, followed by human to human transmission [25], [26], [27], [28]. Serological evidence for henipavirus infection in bats has been reported in the geographic range spanning Australia [29], Malaysia [30], Thailand [31], [32], Cambodia [33], Indonesia [34], Bangladesh [27], India [35], People's Republic of China [36], Papua New Guinea [37], Madagascar [38] and Ghana [39]. Henipaviruses have been isolated from flying foxes in Australia [40], Malaysia [30] and Cambodia [33], and molecular surveys have also identified henipavirus-related RNA in African bats [41].

There are two commercially available bat cell lines, Tb1-Lu (ATCC number CCL-88, derived from the lung of the *Tadarida brasiliensis*) and the Mvi/It (ATCC number CRL-6012, established from an interscapular tumour of *Myotis velifer incautus*). However, neither cell line is susceptible to henipavirus infection (Crameri and Wang, unpublished). Therefore there was a need to establish *Pteropus alecto* cell lines which, coupled with the development of molecular immunological tools, provide the necessary foundation to further investigate infection by henipavirus in its natural reservoir [42]. As fruit bats are implicated as the reservoir of many RNA viruses, such as henipaviruses and many bat rubulaviruses [37], [43], [44], [45], cell lines from this genus may provide new and important insights into virus-host interactions. *P. alecto* cell lines have been established [42] and are a tool that we are using to look at henipavirus infection in bats as a reservoir species.

Fundamental immunological studies on *P. alecto* have been undertaken, including research into the bat toll-like receptors [46], the characterisation of immunoglobulin heavy chain diversity [47] and investigation into type III interferons [48]. The interferon alpha (IFN-α) and beta (IFN-β) genes have been identified in two species of fruit bat, the Egyptian Rousette, *Rousettus aegyptiacus* [49], [50] and the Malaysian flying fox, *Pteropus vampyrus* [51]. The IFN-α gene has also been described in the Greenish Naked-backed fruit bat, *Dobsonia viridis* [52]. The STAT1 protein has also been identified in *R. aegyptiacus* and has the ability to phosphorylate and localize to the nucleus [53]. Similar to that observed in human cells, the function of STAT1 from *R. aegyptiacus* was antagonized during infection with Rabies virus [53]. Bat research to date has provided evidence to suggest that bats have a functional interferon system including signaling pathways that are similar to humans and other mammals.

Investigating the interferon genes and the regulation of innate immunity in bats has previously not been possible due to limited information and a lack of bat-specific immunological reagents. Limited molecular tools have been designed for the *P. alecto* species, and have shown that the interferon production pathway is functional in bat cells [42]. The type I interferons are secreted from most cells in response to virus infection and then bind to the interferon specific receptors in order to activate the expression of numerous interferon stimulated genes (ISGs), many of which have antiviral activities (reviewed in [54]).

In addition to the type I interferons, type III interferons are a relatively recently identified family of interferons that display similar antiviral activity to type I interferons but signal through a distinct receptor complex. The Type III interferon family includes three cytokine members identified in humans: IFN-λ1, IFN-λ2 and IFN-λ3 and are also known as IL-29, IL-28A and IL-28B respectively [55], [56]. In addition, type III interferons have been identified in other mammals, including two IFN-λ genes (IL-29 and IL28B) in *P. alecto* [48]. Recently we reported that *P. alecto* cells infected with the bat paramyxovirus, Tioman virus, are capable of a type III interferon response despite the suppression of type I interferons [48]. Similar to bats, simultaneous induction of type III interferons and suppression of type I interferons have also been reported in human cell lines infected with hantaviruses [57]. As the IFN-λ family of cytokines elicits a similar antiviral response to that of the type I interferons, it would be interesting to know whether the induction of IFN-λ is prevented by the evasion strategies used by henipaviruses (or other viruses), which are known to antagonize the IFN-α/β response.

Most viruses, including the henipaviruses, express one or more interferon antagonist proteins which suppress host interferon production and/or signaling pathways. In this study we have conducted *in vitro* infection studies at biosecurity level 4 (BSL-4), specifically for the purpose of understanding the antagonism of the interferon pathways following henipavirus infection in *P. alecto* cells. We previously reported that interferon production is antagonized in human cells following infection with henipaviruses, while the interferon signaling pathway remains functional [58]. Here we report that henipavirus infection antagonises both interferon induction and signaling in bat cells, thus providing evidence for a significant difference in the antiviral response to henipavirus infection between bats and humans.

## Methods

### Cell culture and viruses

Bat cells used for this study were previously generated from *P. alecto* as described in Crameri *et al* [42]. Cells used include PaLuT02 (clonal *P. alecto* lung cells immortalized with SV40T), PaFe (primary *P. alecto* foetus cells), PaFeT (SV40 T immortalized *P. alecto* foetus cells) and PaKi (primary *P. alecto* kidney cells). All bat cells were maintained in Dulbecco's Modified Eagle's Medium Nutrient Mixture F-12 HAM (Sigma), which also contains 15 mM HEPES, NaHCO3, pyridoxine and L-glutamine, with 10% bovine calf serum (BCS, Hyclone), 100 U/ml penicillin, 100 µg/mL streptomycin and 1.25 ug/ml of amphotericin B (Invitrogen). All cells were maintained at 37°C with 5% CO_2_. Human epidermoid carcinoma cells (HEp-2) were maintained in modified Eagle's Minimal Essential Medium (EMEM) supplemented with 10% BCS (Hyclone), 100 U/ml penicillin, 100 µg/mL streptomycin and 1.25 ug/ml of amphotericin B (Invitrogen).

The henipavirus stocks used in this study were derived from the following isolates: Hendra virus/Australia/Horse/1994/Hendra (HeV), Nipah virus/Malaysia/Human/1999/PKL (NiV-M) and Nipah virus/Bangladesh/Human/2004/Rajbari R1 (NiV-B). All work with live Hendra and Nipah viruses was carried under BSL-4 conditions at the Australian Animal Health Laboratory (AAHL), Geelong, Australia.

### Primer Design

Oligonucleotide primers for amplification of GAPDH, IFN-α, IFN-β, ISG54 and ISG56 were designed from the sequence data of *P. vampyrus* (available in the Ensembl database and the GenBank trace file archive using BLAST on NCBI). BLAST searches and the analysis tools contained within the software package Clone Manager 9 Professional Edition (Scientific & Educational Software, USA) were used to identify and analyse each gene. Nucleotide and protein sequence alignments were performed with sequences from a selection of available mammalian species and used to generate a mammalian consensus sequence. These consensus sequences were used to design oligonucleotide primers to direct the cloning and sequencing of the *P. alecto* genes of interest. The real-time primer sequences for ISG54 are as follows: ISG54F CTACGCCTGGGTCTACTATCAC and ISG54R AATTGCCAGTCCGGAGGAG. The primer sequences for GAPDH, IFN-α and IFN-β [42] and ISG56 [48] have been previously published.

### RNA extraction, reverse transcription and real-time PCR analysis

For interferon production analysis, approximately 0.2×10^6^ cells were infected with a multiplicity of infection (MOI) of 10 TCID_50_/cell with HeV, NiV-M or NiV-B, and at 3 h post infection (pi), the cells were harvested for RNA extraction (RNeasy, Qiagen). For interferon signaling analysis, approximately 0.2×10^6^ cells were infected with HeV, NiV-M or NiV-B (MOI 10). At 24 h pi, the cells were treated with 1000 U of Universal Type I Interferon (PBL). Universal Type I interferon is an IFN-α hybrid constructed from recombinant human IFN-α A and human IFN-α D (Human Interferon α A/D). 3 h post interferon treatment, the cells were harvested, and total RNA isolated. Random primed cDNA was reverse transcribed using Superscript® III Reverse Transcriptase (Invitrogen), and quantitative Real Time PCR was performed using SYBR Green (EXPRESS SYBR® GreenER qPCR SuperMix Universal, Invitrogen) in an ABI 7900 or 7500. The PCR cycling conditions were as follows: one cycle at 95°C for 20 secs and 40 cycles of 95°C for 3 secs and 60°C for 30 secs. Individual mRNA transcripts were assayed in duplicate, and C_T_ values were used to calculate the relative fold changes in each gene. GAPDH mRNA levels were used to normalize samples.

### SDS-PAGE and Western Blot

HEp-2 cells were infected at an MOI of 1 for 24 h, and the PaLuT02 cells were infected at an MOI 10 for 24 h. Cell lysates were prepared and analysed as previously described [58].

### Immunofluorescence

The bat cells (PaLuT02, PaFe, PaFeT, PaKi, PaLuT02) were infected with either HeV, NiV-M or NiV-B at an MOI of 10 for 24 h. Immunofluorescence was undertaken as previously described [58].

## Results

### Viral infection antagonizes interferon production in bat cells

**Figure 1:**
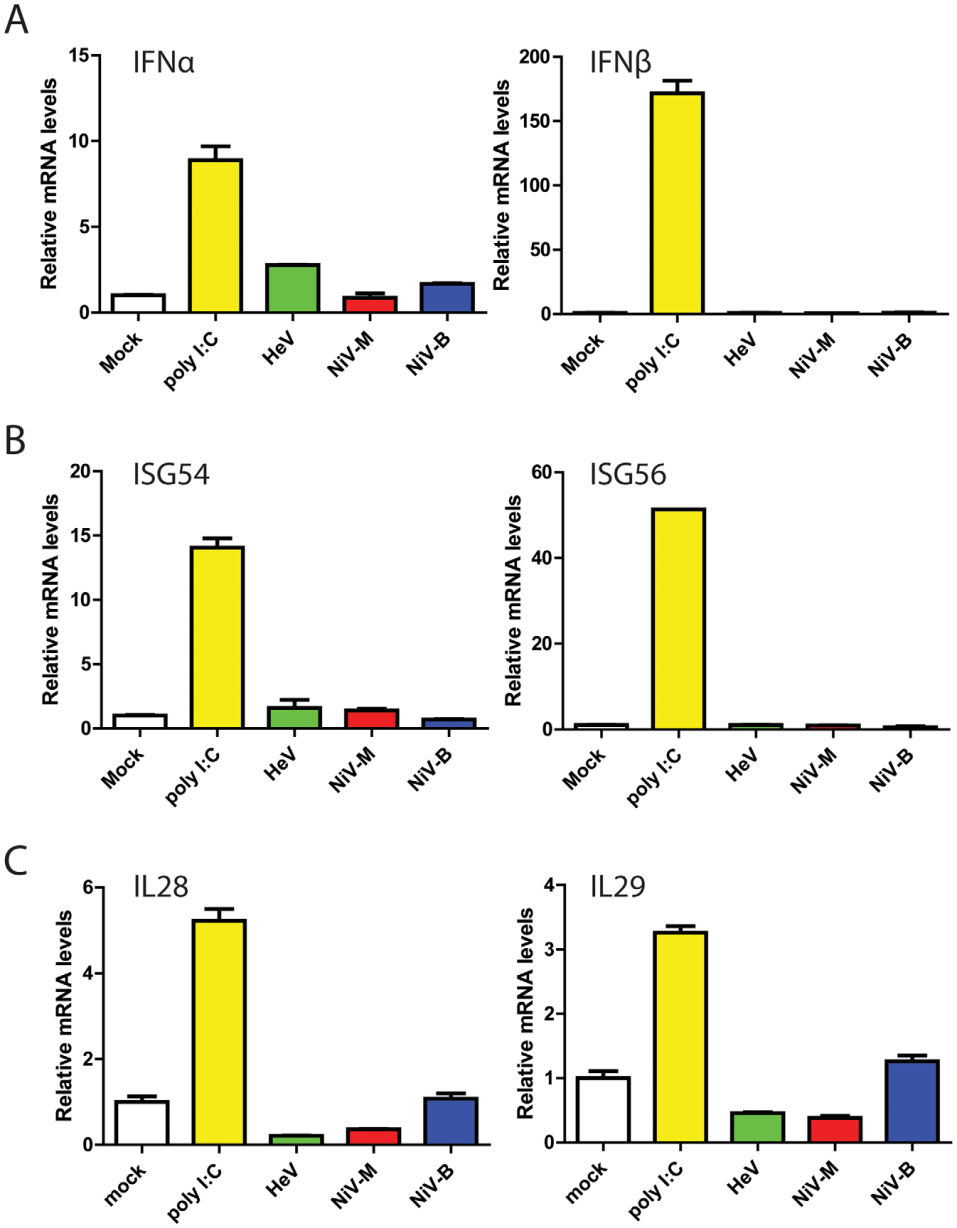
Antagonism of type I interferon production in bat cells infected with henipaviruses. PaLuT02 cells were infected at an MOI of 10 for 3 h. Total RNA was isolated and quantitative real-time PCR was performed using SYBR Green. The (A) IFN-α and IFN-β mRNA levels and, (B) ISG54 and ISG56 mRNA levels were detected and relative fold changes calculated. Transfection of 10 µg poly I∶C was used as a positive control. N = 2 with error bars indicating SEM. (C) IL28B and IL29 mRNA levels were detected and relative fold changes calculated. Transfection of 10 µg poly I∶C was used as a positive control. N = 2 with bars indicating variation between replicates.

Virus infection induces many responses in cells, the most rapid of which include the secretion of type I and III interferons. Stimulation of bat cells with poly I∶C results in the induction of type I (IFN-α and IFN-β) and type III interferons (IFN-λ) [42], [48], [50], demonstrating that the interferon production pathways are functional in these cells. The impact of henipavirus infection on the interferon production pathway in bat lung cells (PaLuT02) was analysed by investigating the regulation of IFN-α and IFN-β mRNA transcripts in comparison to mock infected cells. To examine the effects of henipavirus infection on the interferon production pathway, bat lung cells (PaLuT02) were infected at an MOI of 10 with individual henipaviruses, HeV, NiV-M and NiV-B. After 3 h, total RNA was extracted from cells, and utilized for real-time PCR assays to measure transcriptional regulation of interferon genes. Transfection of 10 µg poly I∶C was used as a positive control as previously reported [42]. Following infection both IFN-α and IFN-β mRNA transcripts were comparable to that observed in mock infected cells (Figure 1a). This suggests that the interferon production pathway is antagonized by each of the three tested henipaviruses, which is comparable to that seen in human cells [58]. The transcriptional regulation of the ISG54 and ISG56 genes was also investigated to confirm that there is a block in interferon production (Figure 1b). The levels of both ISG54 and ISG56 mRNA transcripts were equivalent to mock-infected cells, suggesting that interferon is not produced and therefore no stimulation of the interferon signaling pathway is observed.

In addition to type I interferons, the IFN-λ genes (type III interferons), IL-29 and IL-28B were investigated following henipavirus infection (Figure 1c). Following HeV and NiV-M infection, the IFN-λ transcripts were 50% of the level observed in mock infected cells, strongly suggesting a block in IFN-λ production. The levels of IFN-λ mRNA transcripts following NiV-B infection were comparable with that seen in mock (Figure 1c).

### Viral infection antagonizes the interferon signaling pathway in bat cells

**Figure 2:**
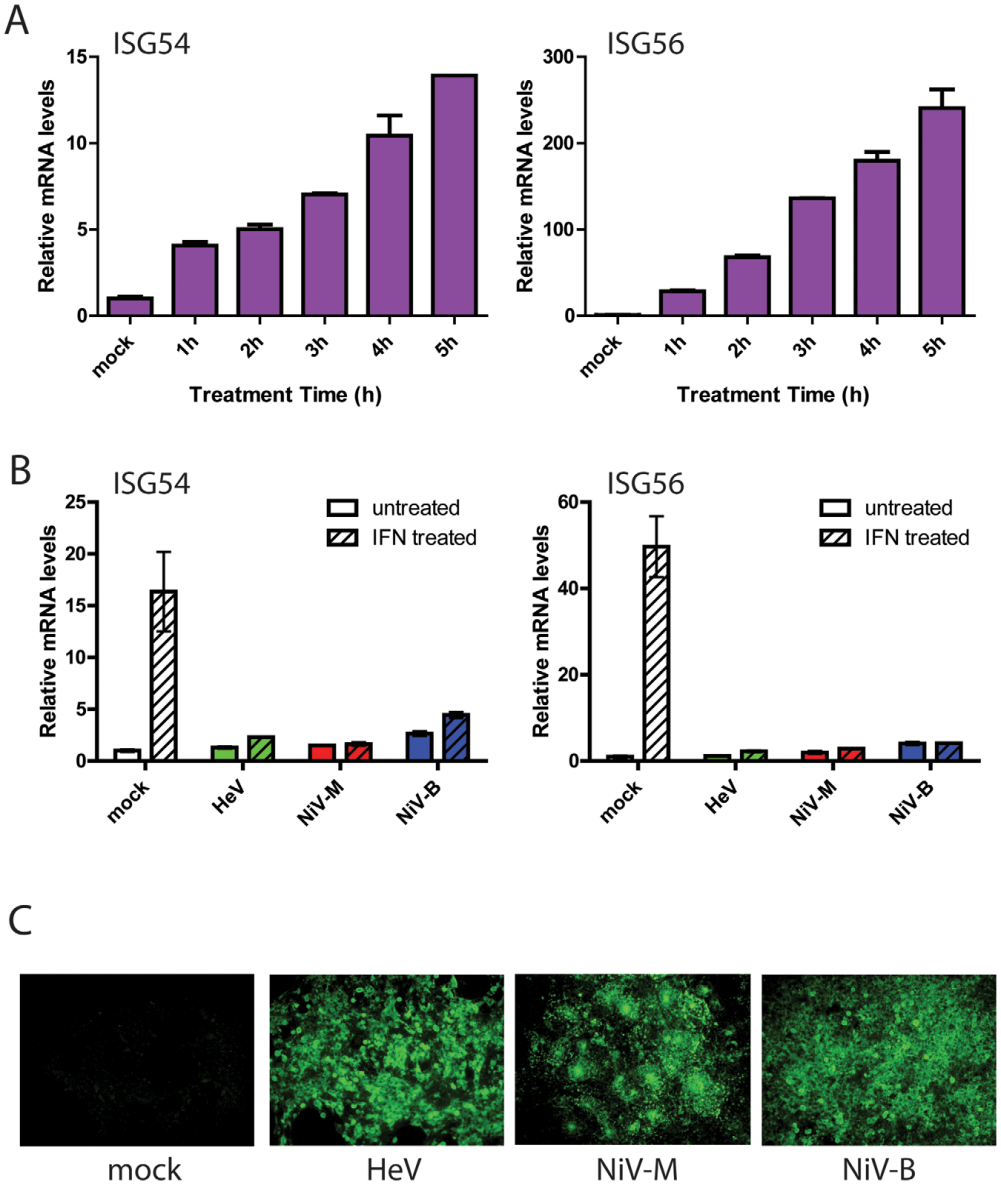
Henipavirus infection and antagonism of the interferon signaling pathway following treatment with exogenous Universal Interferon. (A) PaLuT02 cells were treated with 1000 U of Universal Interferon and cells were harvested at 1 h intervals from 1 to 5 h. Real-time PCR was then performed for ISG54, ISG56. (B) PaLuT02 cells were infected with HeV, NiV-M and NiV-B at an MOI of 10 for 24 h pi, followed by Universal Interferon (1000 U) treatment for 3 h. Real-time PCR was then performed for ISG54, ISG56. The error bars indicate standard deviation of three independent experiments. (C) Immunofluorescent staining of duplicate infections were undertaken to determine level of virus infection using HeV P-specific antisera.

The effect of henipavirus infection on the interferon signaling pathway in PaLuT02 cells was investigated by stimulation with exogenous interferon in order to circumvent the effects of viral antagonism on the interferon production pathway. As there are limited bat immunology reagents available, a universal recombinant human interferon alpha hybrid (PBL) was used. Cells from a variety of mammalian species have been demonstrated to respond to Universal Interferon, including human, monkey, mouse, bovine, rat, cat, pig, rabbit, guinea pig and hamster [59]. Since this product is useful for cross species testing and in cases where autologous interferon is not available, we hypothesized that this interferon would also be effective in bat cells. To confirm activity of Universal Interferon on bat cells, 1000 U of Universal Interferon was used to treat PaLuT02 cells. Upregulation of mRNA transcripts for interferon stimulated genes ISG54 and ISG56 were measured at different time-points. As shown in Figure 2a, an increase in relative ISG54/56 mRNA levels occurred over time, however for practical purposes, a 3 h time-point was chosen for all subsequent experiments. PaLuT02 cells were infected with individual henipaviruses HeV, NiV-M and NiV-B at an MOI of 10 and at 24 h pi the cells were treated with 1000 U of Universal Interferon for 3 h. The cells were harvested for RNA extraction and levels of ISG54 and ISG56 mRNA transcripts were determined. Following interferon treatment of the mock infected cells, the ISG54 and ISG56 were up-regulated by approximately 16-fold and 50-fold, respectively, compared to basal levels (Figure 2b). The untreated HeV, NiV-M and NiV-B infected cells show no up-regulation of ISGs. Following interferon treatment of the infected cells, there is also no significant increase in ISG54 and ISG56 transcripts, demonstrating that interferon signaling is also antagonized following henipavirus infection. Immunofluorescence was undertaken to confirm that the cells were at least 90% infected with HeV, NiV-M and NiV-B prior to undertaking the ISG real-time assays (Figure 2c).

**Figure 3:**
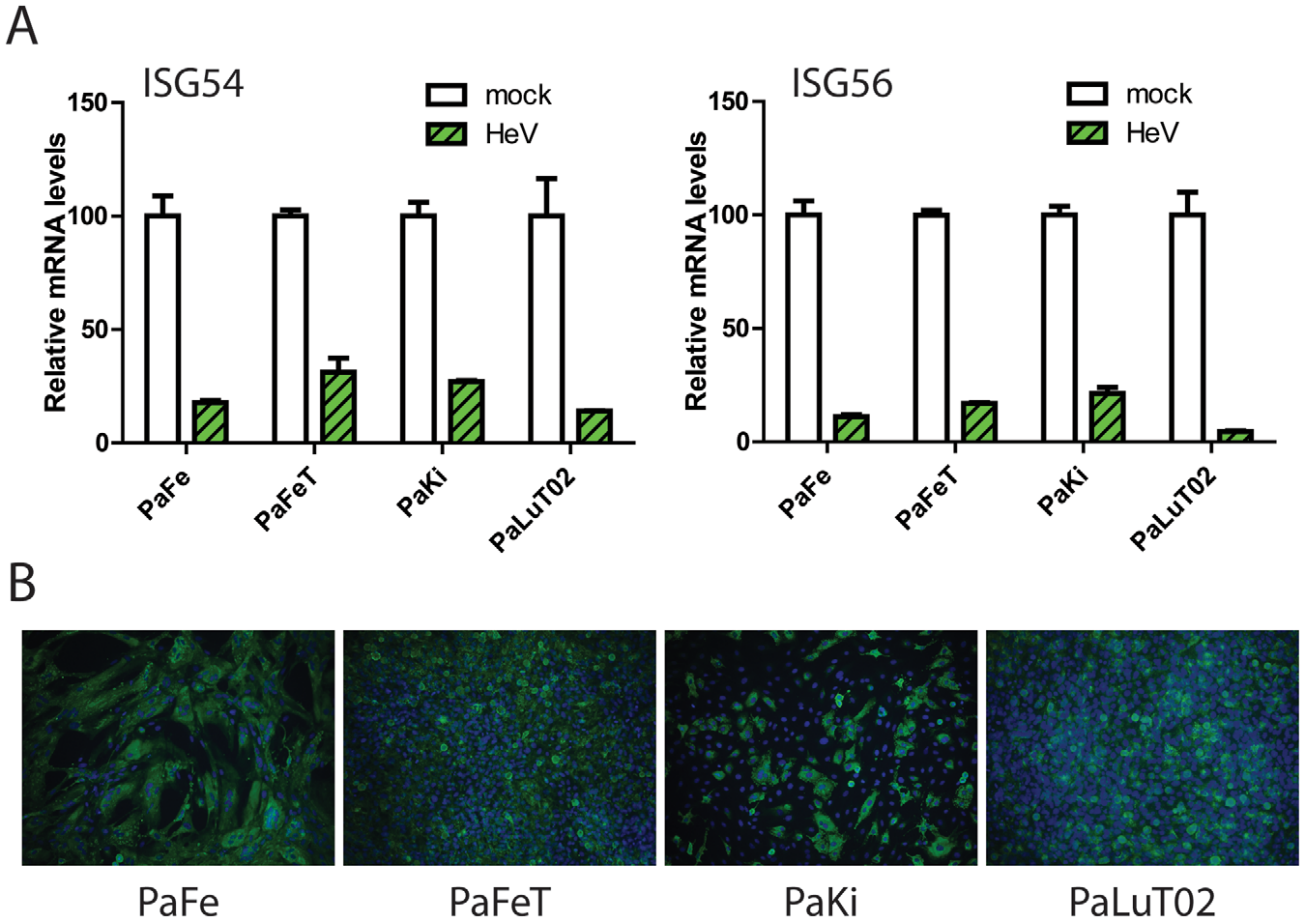
Cell type-independent antagonism of the interferon signaling pathway in bat cells. (A) PaFe, PaFeT, PaKi and PaLuT02 cells were infected with HeV at a high MOI for 24 h pi, followed by treatment with Universal Interferon (1000 U) for 3 h. Real-time PCR was then performed for ISG54 and ISG56. N = 2 with error bars indicating SEM. (B) Immunofluorescent staining of duplicate infections were undertaken to determine level of virus infection using HeV P-specific antisera.

Due to the differences observed following henipavirus infection and interferon signaling in bat cells compared to human cells, the experiment was repeated in several different *P. alecto* cell lines. Cells were infected with HeV in order to determine whether a block in interferon signaling is universal or unique to the PaLuT02 cells. Primary fetus and kidney cells (PaFe and Paki), and cloned, immortalised fetus and lung cells (PaFeT and PaLuT02) were infected with HeV for 24 h at a high MOI and were treated with Universal Interferon (1000 U) for 3 h prior to harvesting for real time assays (Figure 3a). A percentage block in ISG54 and ISG56 induction was calculated with mock-infected cells being set at 100% for each cell type. In all cell types there was at least a 75–80% reduction in ISG54/56 transcription compared to mock-infected cells (Figure 3a). Immunofluorescent detection of duplicate HeV infected cells confirmed a 75–80% level of infection (Figure 3b).

### Comparable levels of henipavirus proteins are expressed in bat and human cells

**Figure 4:**
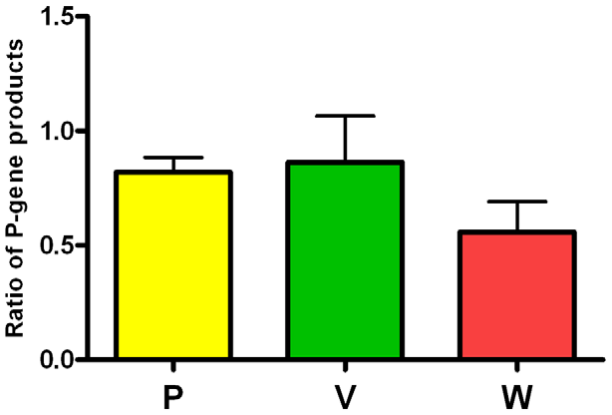
Level of expression for henipavirus P-gene products in *P. alecto* and human cells. *P. alecto* and HEp-2 cells were infected at an MOI of 10 or 1 respectively for 24 hours then lysed with 2% SDS to inactivate virus prior to removal from the BSL4 laboratory. The cell lysates were resolved by 12% SDS-PAGE and detected by Western blotting with rabbit antiserum specific for the N protein and the unique C-terminal portions of the P, V and W proteins. The C-terminal P, V and W protein antisera were generated against synthesized peptides (Genscript Pty Ltd). Levels of protein were semi-quantified using ImageJ software [70] and relative protein levels normalized to N protein were calculated for each sample. The bars represent ratio of protein expression in *P. alecto* cells over human cells using data generated from four independent infection experiments, with error bars indicating SEM.

Recently we reported the interferon response of human cells to infection with henipavirus compared with transfection with NiV P, V and W gene products. [58]. We hypothesized that the amount of viral protein expressed may dictate the level of antagonism observed on the interferon production and signaling pathways. Due to the apparent differences in interferon signaling antagonism displayed by human and bat cells infected with henipaviruses, we investigated the protein expression levels of the P-gene products in the two host cell types. SDS-PAGE and Western blot analysis were performed on multiple cell lysates to investigate the ratio of the P-gene products (P, V and W) relative to N protein expression. The lysates prepared from human (HEp-2) and *P. alecto* (PaLuT02) cells showed a decreased level of P, V and W expression in *P. alecto* cell lines compared with human cells (Figure 4). The results suggest that the block in interferon signaling in *P. alecto* cells is not a result of differential expression of P-gene products.

## Discussion

Recently we described the interferon response of human cells to henipavirus infection, demonstrating a block in the induction of the interferon production pathway, with little effect on interferon signaling (Virtue, 2011). This contrasted with that reported from transfected cells expressing P gene derived proteins [60], [61], [62], [63], [64], [65]. We further demonstrated that transfection of cells with henipavirus P-gene products leads to a significant increase in the level of protein expression in cells and, we hypothesized that the different results could be accounted for by viral protein levels [58]. In henipavirus infected bat cells, the interferon production pathway was antagonized, similar to that seen in human cell lines [58]. In addition to type I interferons, the recently identified type III interferons were also suppressed in henipavirus infected bat cells. This result contrasts with the up-regulation of type III interferons in bat cells infected with another bat paramyxovirus, Tioman virus. It was previously hypothesized that the type III interferons may represent an alternative antiviral strategy following suppression of type I interferons [48]. However, these results demonstrate that this does not appear to be the case during henipavirus infection.

In addition to suppression of interferon production, the interferon signaling pathway was also antagonized in bat cell lines following infection as demonstrated by the absence of an ISG response. This block in interferon signaling was observed in multiple cell lines derived from several different organs. Although the block is not 100% following HeV infection, we hypothesize that this is due to a small percentage of uninfected cells, and not leaky activation of the interferon signaling pathway. This hypothesis is reflected in the level of infected cells seen by immunofluorescence on duplicate samples at the point of RNA extraction. The antagonism of the interferon signaling pathway in all cell types suggests that the block in interferon signaling is not cell type specific, and is a universal response that would be expected to occur *in vivo* in bats. Following this study, investigation into the effect of henipavirus infection on immune cells, such as dendritic cells, will also be undertaken.

Although interferon induction in human cell lines is inhibited by henipavirus infection, treatment with exogenous interferon restores the ISG response [58]. In contrast, exogenous interferon failed to induce an ISG response in henipavirus infected bat cells, consistent with a block in both interferon induction and signaling pathways in bat cells. This difference was not due to different ratios of henipavirus P-gene products expressed in bats cells compared to human cells. Therefore, we conclude that the block in interferon signaling in bat cells is not due to an increase in total P gene product expression or an altered ratio of P/V/W in cells, but due to an as yet unidentified factor. Potentially this difference could be due to increased binding affinity of the P gene products to the *P. alecto* STAT1. We previously determined that henipavirus proteins only antagonise the interferon signaling pathway in human cells as an artefact of overexpression systems, not during *in vitro* infection studies [58]. However interaction of P/V/W with STAT1 in human cells has been identified as the mechanism for the antagonism of the interferon signaling pathways during single gene studies [66], [67], [68], [69].

These results highlight a difference in henipavirus-host interactions between bats (natural reservoir host) and humans (spill-over host, with a fatal outcome). Transcription and expression of ISGs results in the generation of an antiviral state in cells, preventing infection and replication of virus in neighbouring cells. Blocking both the interferon production and signaling pathways is the optimal mechanism for a virus to counteract the host response. If the production pathway is blocked, no interferon is secreted. However, professional antigen presenting cells such as dendritic cells are programmed to produce large amounts of interferon, which may not be prevented by viral interferon antagonist proteins. Blocking the signaling pathway in this situation is insurance, as the virus can block the effect of interferon. Blocking both pathways is considered to give the virus a strong advantage over the host and would be expected to allow for improved infection in the host where this is possible. The ability of henipavirus to block interferon induction and signaling in bat cells is surprising given the asymptomatic nature of henipavirus infection in bats compared with humans, which have an intact signaling response but generally fatal response to infection. This would suggest that bats control henipavirus infection by an as yet unidentified mechanism, not via the interferon response. Given the importance of the interferon response in controlling viral replication in other mammals, this result is significant, providing evidence for differences in the antiviral response of bats compared to humans.

In this study, we have shown that henipaviruses block interferon production in bat cells. We have also demonstrated differences in the ability of the henipaviruses to block the interferon signaling pathway in bat cells compared with human cells and this block is not due to an increased level of viral protein expression. We hypothesize that the interferon response is not responsible for the differences in the susceptibility of bats and humans to henipavirus infection. Identification of the mechanism by which bats control viral infections has the potential to direct research in the development of new, broadly active antiviral strategies. This study has added to the reported investigations into virus-bat interaction and bat immunology in general.
